# Preliminary Validation of the Children’s Auditory Performance Scale (CHAPS) and the Auditory Processing Domain Questionnaire (APDQ) in Greek Cypriot Children

**DOI:** 10.3390/audiolres14040053

**Published:** 2024-07-15

**Authors:** Konstantinos Drosos, Dionysios Tafiadis, Louiza Voniati, Alexandra Papanicolaou, Chryssoula Thodi

**Affiliations:** 1 School of Sciences, Speech and Language Therapy, European University Cyprus, 2404 Nicosia, Cyprus; l.voniati@euc.ac.cy (L.V.); c.thodi@euc.ac.cy (C.T.); 2 Department of Speech and Language Therapy, University of Ioannina, GR-45500 Ioannina, Greece; tafiadis@uoi.gr; 3 Department of Hearing and Speech Sciences, University of Maryland College Park, College Park, MD 20740, USA; apapani3@umd.edu

**Keywords:** auditory processing, discrimination, screening, CHAPS, APDQ, speech sound disorders

## Abstract

Background: Identification of auditory processing disorders is achieved using questionnaires along with linguistic, non-linguistic, and auditory processing tests. Notably, the questionnaires “Children’s Auditory Performance Scale” (CHAPS) and “Auditory Processing Domain Questionnaire” (APDQ) are widely recognized and used. The current study investigated the psychometric properties of the CHAPS and APDQ in Greek Cypriot children. Methods: The CHAPS and APDQ questionnaires were completed by parents of 40 Greek Cypriot children, 16 typically developing (TD) children, and 24 children with a history of Speech Sound Disorders (SSDs). Results: There were significant differences between the two groups on both questionnaires. Cronbach’s alpha was calculated at α = 0.922 for the CHAPS total score and α = 0.926 for the APDQ total score. The Receiver Operating Curve (ROC) analysis provided a cut-off point equal to −0.30 (AUC 0.849, *p* < 0.001) for CHAPS and a cut-off point equal to 90.00 (AUC 0.820, *p* < 0.001) for APDQ. Significant positive Spearman ρ correlations were observed between the CHAPS and APDQ (ρ = 0.639, *p* = 0.001). Conclusions: The CHAPS and APDQ can identify distinct auditory processing characteristics between in children with SSDs and TD children.

## 1. Introduction

Auditory processing disorder (APD) refers to a deficit in the neural processing of auditory information that can result in higher-order disorders related to learning, attention, memory, cognitive, communicative, or language-related skills [1,2]. APD affects an individual’s ability to adequately acquire auditory skills (i.e., detection, discrimination, identification, and comprehension of speech), especially in complex noisy listening environments [1,3]. In children, the estimated prevalence is between 0.2% and 5% [4,5,6] noting a 2:1 male-to-female ratio of APD diagnosis. Several studies suggest high comorbidity between APD and developmental disorders such as developmental language disorder (DLD), dyslexia, learning disabilities (LDs), attention-deficit hyperactivity disorder (ADHD), autism spectrum disorders (ASD) [7,8,9], and Speech Sound Disorders (SSDs) [10]. Recognizing auditory processing impairments in children early on is crucial for implementing successful auditory interventions. This takes advantage of the brain’s adaptability and allows for accurate differentiation of children with APD from those with related disorders [8]. It is essential to gather detailed reports on a child’s listening difficulties in everyday settings to identify children who may be at risk for suspected APD. This can be achieved by administering screening questionnaires to caregivers and teachers [11,12]. The American Academy of Audiology (AAA) [3] and the American Speech–Language–Hearing Association (ASHA) [1] recommend employing screening tools to pinpoint those who may be susceptible to APD. Questionnaires and behavioral checklists that have been mentioned in the international literature include the following: (1) the Children’s Auditory Performance Scale (CHAPS); (2) the Screening Instrument for Targeting Educational Risk (SIFTER); (3) the Test of Auditory Processing Skills–Revised (TAPS-R); (4) the Children’s Home Inventory of Listening Difficulties (CHILD); (5) Fisher’s Auditory Problems Checklist (FAPC); (6) the Auditory Processing Domains Questionnaire (APDQ); (7) the Listening Inventory for Education (LIFE); (8) the Listening Inventory for Education–Revised (LIFE-R); (9) the Scale of Auditory Behaviors (SAB); (10) the Listening Inventory (TLI); and (11) the Evaluation of Children’s Listening and Processing Skills (ECLIPS). Worldwide, researchers are continuously evaluating the use of APD screening tools and questionnaires based on relevance and clinical applicability [10,11,12,13,14]. Children with SSDs, as well as children with suspected APD, may have differences in auditory processing skills from TD children and children with APD diagnosis [8,10,15]. A crucial effort of this study was to reflect findings from the available literature on the population of children in Cyprus. Hence, we proceeded to administer the CHAPS and APDQ to record the auditory processing skills of Greek Cypriot children with typical development and children with SSDs. Therefore, this study examines the effectiveness of two established and commonly utilized questionnaires, the CHAPS and APDQ [12,16,17,18], in detecting and differentiating children with APD from TD children and children with SSDs. Additionally, this study aims to evaluate the suitability of these questionnaires as screening tools in Greek Cypriot children. The two questionnaires chosen for the present study are commonly accepted by the scientific community, are bibliographically recorded internationally in the existing knowledge of systematic reviews on the subject, and offer detailed results with relative subdomains covering audiological, linguistic, and non-linguistic fields [11,12].

## 2. Materials and Methods

### 2.1. Sample

The parents of 40 children between seven and ten years old filled out the CHAPS and APDQ questionnaires. Sixteen children who exhibited typical psychosocial and language development, of average age 8.40 years (7.85–8.70 years), formed the typically developing (TD) group. Twenty-four children diagnosed with Speech Sound Disorders (SSDs), of average age 8.1 years (7.90–8.40 years) formed the experimental group. All children were monolingual Greek Cypriot dialect speakers. Children in both groups had normal pure-tone thresholds, no history of middle ear disease, and no sensory, cognitive, psychomotor, communication, or learning disorders.

### 2.2. Translation Procedures of APDQ

The APDQ questionnaire was translated and adapted following the Cognitive Debriefing Method as outlined by Hall et al. [19]. Following communication with the original creators of the APDQ questionnaire, we proceeded to translate and adapt the questions to the Greek language. Initially, two native speakers of Greek proficient in English translated the questionnaire from English to Greek. A reconciliation version of the two translations was developed with the mediation of a third native Greek Speaker fluent in English. Following the initial stage, a proficient English Speaker back-translated the questionnaire to English, and the points showing discrepancies with the original were reworked in the translation to Greek. Following a consensus review of the two procedures, the form was finalized. The questionnaire was administered to three parents to ensure feasibility. Upon feedback from the pilot study, the team finalized the questionnaire.

### 2.3. Participant Recruitment and Assessment

The recruitment process and consent form were approved by the Cyprus National Bioethics Committee (EEBK/EP/2022/37). Invitations to participate in the study were extended to all parents of children undergoing therapy for SSDs at the Speech, Language, and Hearing Clinic of the European University Cyprus. The parents who agreed to allow their children to participate completed and signed the consent form. Parents provided information on their children’s health history. TD children were recruited from public schools. The inclusion of children in public schools was approved by the Pedagogical Institute of the Ministry of Education, Sport, and Youth of the Republic of Cyprus ΚΕΕA-148053. A pure-tone audiogram was completed by an audiologist at the Speech, Language, and Hearing Clinic of the European University Cyprus.

Following audiometric testing, the children completed an auditory processing battery comprising the Gap Detection Test [20]; Gap-in-Noise Test [21]; Dichotic Hearing (words) [18]; Greek Speech in Bubble Test [22]; Duration and Pitch Pattern Sequence Tests [23]; Forward/Backward digit span [24]; and words presented in various signal-to-noise ratios. Children completed a battery of speech and language tests, consisting of the Action Picture Test (APT) [25] and the Metaphone test–full version (Test of Metaphonological Development and Reading Readiness in Phonological Awareness) [26]. The APT assesses the content and grammatical patterns in children’s speech, effectively documenting the morphology in terms of grammar and syntax. The test considers grammatical structures and words used to convey information. The Metaphone test evaluates linguistic abilities related to rhyme, syllabic, and phonemic awareness. The Raven Colored Progressive Matrices Assessment (Raven) [27] was included in the protocol as an index of cognitive performance. The Raven is a non-verbal assessment of abstract and cognitive function, spatial reasoning, analogical reasoning, and problem-solving ability.

Parents of participating children filled out the CHAPS and APDQ questionnaires, which aimed to document the auditory processing profiles of the children and identify any distinguishing factors between the two groups.

The CHAPS questionnaire collects caregivers’ and teachers’ feedback on children’s hearing difficulties at home and in academic settings [18,28]. The CHAPS includes thirty-six questions divided into six subscales: (1) perception in noise; (2) perception in quiet; (3) perception in ideal (one-on-one) situations; (4) perception with multiple inputs; (5) memory; and (6) attention. The first four subscales directly assess hearing-related difficulties, while the last two evaluate cognitive skills indirectly related to hearing. Responders are asked to rate the child’s hearing difficulties relative to their peers using the following Likert scale: less difficulty (+1), same amount of difficulty (0), slightly more difficulty (−1), more difficulty (−2), considerably more difficulty (−3), significantly more difficulty (−4), and cannot function at all (−5). A raw score is calculated by adding the scores of individual question items; the overall result is divided by 7 to obtain an average overall score. Raw scores are available for each subscale; when divided by the number of questions, we obtain an overall score. The CHAPS questionnaire is utilized to screen and complement the diagnostic auditory processing evaluation. Silva et al. [29] published a systematic review. They investigated the accuracy of screening instruments for identifying APD and determined that the sensitivity of the CHAPS was higher than 70% among reviewed articles. In our research study, we used the Greek version of the CHAPS [18].

The APDQ was developed by O’Hara and Mealings in 2018 and revised in 2021 [16]. In the current iteration, the APDQ contains fifty questions divided into three sub-scales: (1) the Auditory Processing (AP) scale; (2) the Attention Control (ATT) scale; and (3) the Language (Lang) scale. One question overlaps between the AP and ATT scales, one question overlaps between the AP and Lang scales, and two questions are used to compare listening in quiet versus listening in noise. A behavior is attributed four points if it is performed regularly (>3/4 of the time), three points if it is performed often (1/2–3/4 of the time), one point if it is performed sometimes (1/4–1/2 of the time), and zero points if it is performed rarely (<1/4 of the time). Scale scores are calculated as the sum of item scores per scale divided by the number of items in each scale multiplied by four. Scale scores are reported as “percent of perfect score”: scores above 75% are interpreted as greater skill competency, whereas scores below 25% are interpreted as reduced skill competency. Additionally, the APDQ includes an algorithmic indicator called Target AP, which is derived by subtracting the Auditory Processing domain score from the Attention domain score.

### 2.4. Statistical Analysis

Variables were evaluated for normality using the Kolmogorov–Smirnov and Shapiro–Wilk tests; descriptive variables reported included the Median (Mdn) and Interquartile range (IQR). The Mann–Whitney test was used to investigate differences between the two independent groups. A size effect analysis was performed with a partial eta squared coefficient for non-parametric values (n^2^). The estimation of cut-off values for the CHAPS was computed through a Receiver Operating Characteristics (ROC) curve analysis. The optimal cut-off values were determined using the robust and effective Youden index. The Cronbach’s alpha coefficient technique was used to evaluate the internal consistency of the Greek version of the two questionnaires.

To determine questionnaire sensitivity and specificity, we computed Spearman’s rho between the total scores of the CHAPS, the APDQ, and the auditory processing scores.

The significance level was set at 0.05 for two-tailed comparisons. Statistical analyses were performed using IBM SPSS 28 statistical software [30].

## 3. Results

### 3.1. Demographic and Group Performance Findings

TD and children with SSDs had similar age and cognitive characteristics. Participants’ demographics, language, and language performance are shown in Table 1. Bonferroni’s adjustment for multiple comparisons was applied on the acceptable α level for the subdomain comparisons of the Action Pictures Test; for four comparisons, the acceptable level would be 0.05/4 = 0.0125. For the Metaphone test subdomain comparisons, the acceptable level would be 0.05/9 = 0.0056. Table 2 shows the auditory processing battery findings.

### 3.2. CHAPS Questionnaire and APDQ Findings

Table 3 shows the group performance and comparisons on the CHAPS questionnaire. The CHAPS questionnaire results indicate that the experimental group had significantly higher difficulty than the TD group [U = 1.500, *p* < 0.001] in all six CHAPS subdomains. Bonferroni’s adjustment for multiple comparisons was applied on a level for the subdomain comparisons of the CHAPS test; for six comparisons the acceptable level would be 0.05/6 = 0.008. Table 4 shows the group performance and comparisons on the APDQ questionnaire There was a significant difference between the groups on the APDQ total score [U = 49.000, *p* = 0.001] and on the APDQ subdomains.

### 3.3. Receiver Operating Characteristics (ROC) Analysis for CHAPS and APDQ Questionnaires

#### 3.3.1. ROC Analysis for CHAPS Questionnaire

**Figure 1:**
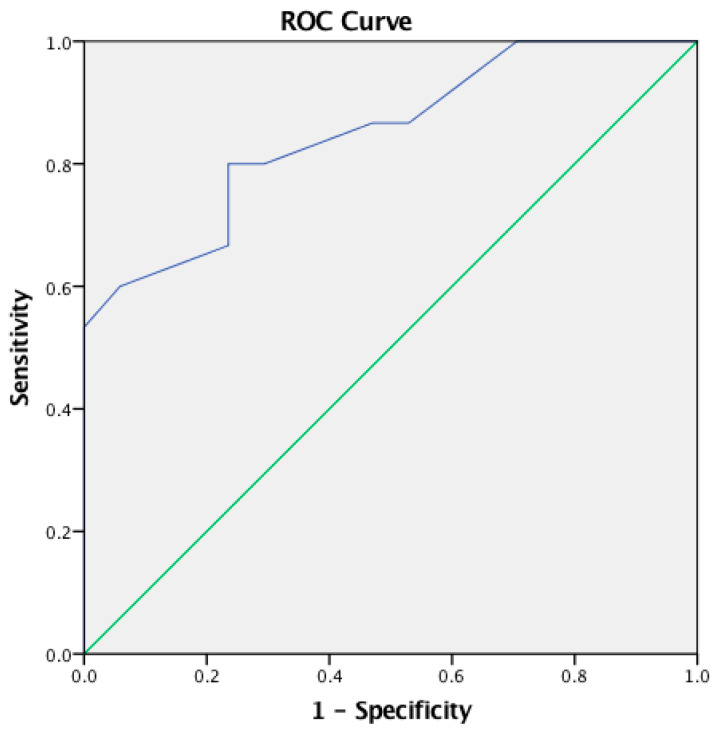
Receiver Operating Characteristics (ROC) curve for the CHAPS questionnaire total score—between parents of experimental group and parents of TD children.

The cut-off point of the CHAPS questionnaire total score was computed using ROC analysis. There was positive discrimination between children in the experimental group and TD children [AUC 0.849 (95% CI: 0.715–0.983), *p* = 0.001]. According to the Youden Index, the optimal cut-off point was −0.30 with a sensitivity of 0.800 and specificity of 0.765 for the CHAPS questionnaire total score (see Table 5; Figure 1).

**Figure 2:**
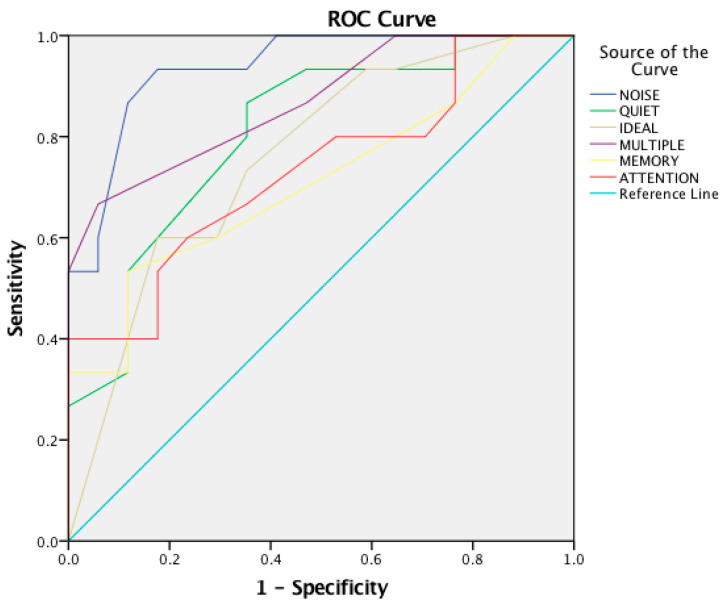
Receiver Operating Characteristics (ROC) curve for the six subdomains of CHAPS questionnaire—between parents of experimental group and parents group of TD children.

Additionally, an ROC analysis revealed a positive discrimination for all CHAPS total score and CHAPS six subdomains as shown in Table 5 and Figure 1 and Figure 2.

#### 3.3.2. ROC Analysis for APDQ Questionnaire

**Figure 3:**
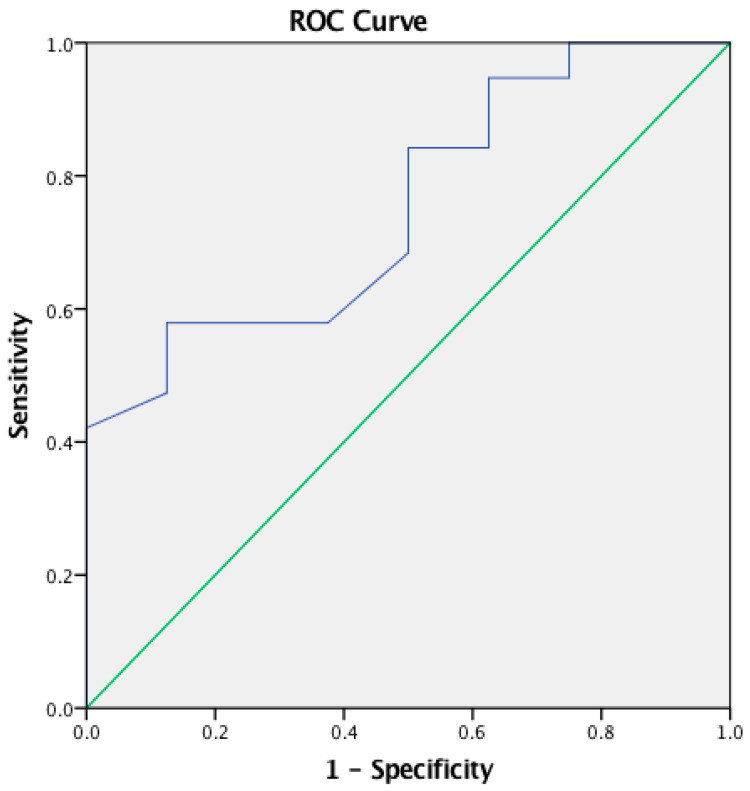
Receiver Operating Characteristics (ROC) curve for the APDQ questionnaire total score—between parents of experimental group and parents of TD children.

The cut-off point of the APDQ questionnaire total score was computed using an ROC analysis. There was positive discrimination between children in the experimental group and TD children [AUC 0.820 (95% CI: 0.679–0.961), *p* < 0.001]. The optimal cut-off point was 90.00 with a sensitivity of 0.647 and 1-specificity of 0.750 according to the Youden Index for the APDQ questionnaire total score (see Table 6; Figure 3).

**Figure 4:**
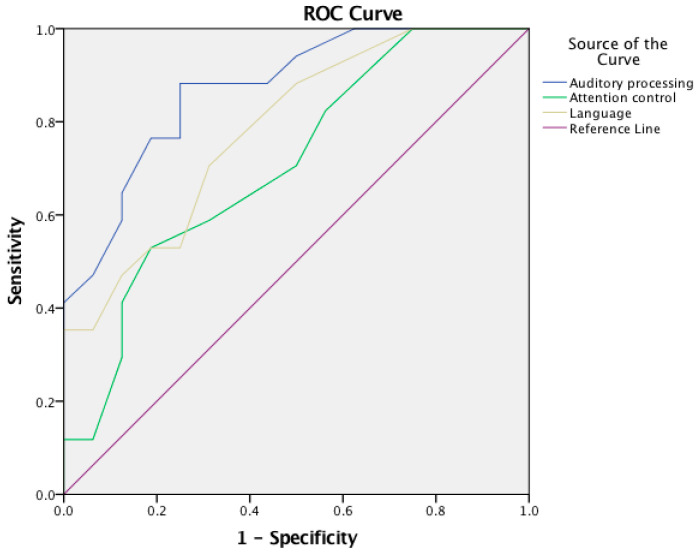
Receiver Operating Characteristics (ROC) curve of APDQ questionnaire—between parents of experimental group and parents of TD children for the APDQ Auditory Processing, Attention, and Language factors.

**Figure 5:**
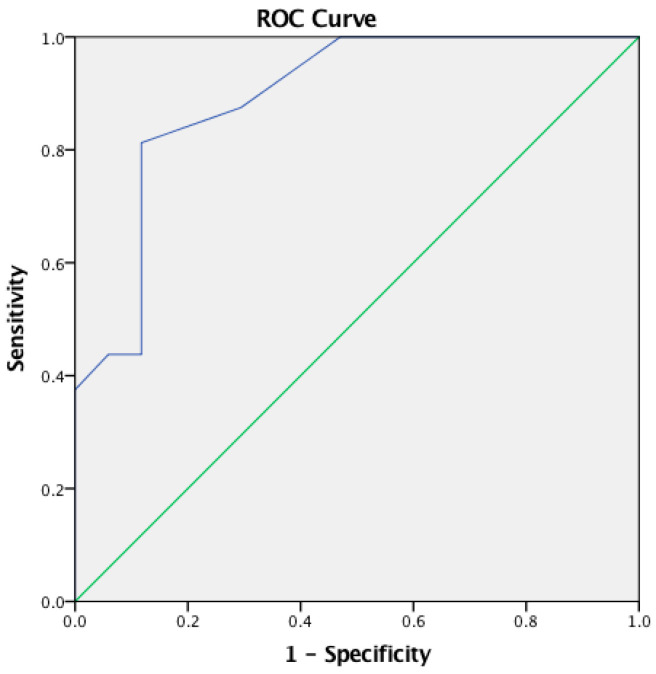
Receiver Operating Characteristics (ROC) curve of APDQ questionnaire—between parents of experimental group and parents of TD children for the APDQ Auditory Processing Target subdomain.

An ROC analysis revealed significant positive discrimination for the three APDQ subdomains as shown in Table 6 and Figure 4 and the APDQ Auditory Processing Target subdomain in Figure 5.

### 3.4. Reliability and Validity Measures for CHAPS and APDQ Questionnaires

The CHAPS total score internal consistency was excellent (Cronbach’s alpha α = 0.922). The item scale correlations of the CHAPS six items ranged from 0.896 to 0.930. Similarly, an excellent internal consistency was estimated for the APDQ total score (Cronbach’s alpha α = 0.926), with item scale correlations of APDQ 50 items ranging from 0.922 to 0.931.

The Spearman ρ correlations were computed between the Greek versions of the CHAPS Total Score and the APDQ for determining the sensitivity against an external validity criterion and the analysis returned a statistically significant positive result (ρ = 0.639, *p* = 0.001).

APD is a multifactorial process that requires audiological, linguistic, and non-linguistic as well as electrophysiological tests [12,22,29]. Correlations between CHAPS and APDQ questionnaire scores and auditory processing performance provide external validation for the capacity of the questionnaires to identify APD. The statistically significant correlations are presented in Table 7.

## 4. Discussion

The current study investigated auditory processing characteristics among Greek Cypriot children aged 7–10 years (typically developing and with SSD), as reflected by the CHAPS and APDQ questionnaires. Findings highlight the capacity of the questionnaires to differentiate between the two populations in terms of auditory processing and explore correlations between questionnaire indices and auditory processing performance. A recent systematic review indicated that APD may be a major underlying factor in children with SSD, as lower scores on auditory processing tasks are often observed in children with varying SSD [10,29]. Kalnak and Nakena von Mentez [31] reported that children with phonological disorders have significant auditory processing and auditory sensitivity difficulties compared to children with typical development.

Our findings align with previous reports and underscore the utility of CHAPS and APDQ as effective tools for identifying listening difficulties. We concur with the recommendations that questionnaires can be used to identify children at risk for auditory processing disorders (APD) across diverse populations, as supported by the literature [10,11,12,15,18,31,32,33]. Furthermore, our findings add the SSD population to the clinical groups that might benefit from the identification of auditory processing through the CHAPS and APDQ questionnaires [11,12].

### 4.1. CHAPS Questionnaire

The CHAPS can distinguish between typical and clinical populations as it successfully differentiated children with suspected APD [34] from typically developing children of similar age to our study [35]. It was also effective in distinguishing children with listening difficulties from typically developing children in the older age range of 7–12 years [28]. Additionally, the CHAPS identified listening difficulties in children diagnosed with ADHD [35]. The CHAPS successfully discriminated typically developing Greek children from children diagnosed with APD at the age of 12 years [18]. Our results indicated that the CHAPS can be used to distinguish auditory processing characteristics between TD children and children with SSDs: children in our SSD group had lower scores in the total scores in all subdomains of the CHAPS. Our findings further corroborate previous reports [28,32,34,35].

Notably, ours is the first study to specifically investigate children with SSDs and compare their performance on the CHAPS and APDQ to the performance of typically developing children. Our findings indicate that the CHAPS can effectively differentiate children with listening difficulties or a lower auditory processing profile, placing them in a high-risk category for APD. The CHAPS may guide specialists to refer children for a comprehensive AP evaluation when these difficulties are identified. The use of CHAPS as a screening tool has been discussed in prior research reports [11,12,18,28,32,34,35,36].

#### 4.1.1. CHAPS ROC Analysis

There is a paucity of reported cut-off points using the area under the curve (AUC) from ROC analysis. The two reports with cut-off points are Ahmmed and Ahmmed’s [34] report on children with suspected APD, 6–11 years, and Sanchez and Lam [36], who report on children diagnosed with ADHD 7–10 years of age. Sanchez and Lam’s study reported cut-off points with no statistical significance, rendering comparisons quite difficult [36]; however, the study is included in Table 8 to be exhaustive in our report.

Ahmmed and Ahmmed [34] established specific cut-off points for children with suspected APD by determining whether the children’s performance fell one standard deviation lower (SD), 1.5 SD lower, or 2 SD lower than the norm. They further evaluated the likelihood of an APD diagnosis based on the criteria outlined by the ASHA [1] and the AAA [3] guidelines. Cut-off values were not reported for the “Noise” subdomain, the “Quiet” subdomain, the “Multiple inputs” subdomain, and for the overall performance. Our cut-off points are quite different from Ahmmed and Ahmmed’s [34] study, with slightly higher values overall; differences can be attributed to the clinical populations assessed and the method of cut-off point estimation. Their study parallels our findings in that both clinical populations had indications of listening difficulties as identified by CHAPS.

Sanchez and Lam’s [36] results differ from ours as a whole and in the given subdomains: they included children with a diagnosis of ADHD and co-morbidity of learning difficulties. Children with ADHD may have a different auditory processing profile compared to children with typical development or children with comorbid learning difficulties.

Despite variations arising from different populations, age groups, cultural characteristics, and diagnoses, the specified cut-off points have consistently distinguished study populations from typically developing individuals. It is essential to consider these indicators alongside comprehensive assessments of linguistic and non-linguistic test results and auditory processing evaluations.

#### 4.1.2. CHAPS Psychometric Properties

The CHAPS psychometric properties we report here concur with related international studies; most importantly, our findings agree with the Greek research by Iliadou and Bamiou [18] and others [17,28,34,36,37]. Similarly, the current study’s intraclass classification coefficient Cronbach’s alpha (α = 0.922) was excellent. Our findings further documented that the CHAPS is a reliable and valid index questionnaire for screening and identifying auditory processing difficulties in the Cypriot Greek population.

### 4.2. APDQ Questionnaire

The APDQ has successfully differentiated clinical populations from typically developing children and effectively identified distinct auditory processing characteristics in children diagnosed with APD, ADHD, and learning disabilities [16]. Similarly, we found that the total scores and measurements in the four subdomains of the APDQ were poorer in children with SSDs compared to typically developing children. Our study has confirmed that the APDQ can effectively differentiate the auditory processing difficulties of children with SSDs from those of typically developing children. These findings underscore the clinical value of the APDQ.

#### 4.2.1. APDQ ROC Analysis

To date, only Ahmadi et al. [38] have reported cut-off points for the APDQ. They indicated cut-off values of 76.20 for auditory processing, 80.92 for target auditory processing, 78.40 for language, and 61.25 for attention, based on the differentiation between children diagnosed with APD and children diagnosed with APD and learning difficulties. The authors did not detail the methodology they followed, nor did they estimate the overall cut-off point. The cut-off values they reported related to children diagnosed with APD and do not compare with a typically developing population. We have found significantly lower APDQ performance and cut-off levels from Ahmad et al. [38]; this finding can be attributed to the significantly younger age of our sample (children aged 7–10 years), whereas Ahmadi et al. evaluated children aged 7–17 years. Population variations notwithstanding, children with comorbid conditions would be expected to have poorer auditory processing performance [8,10,32]. Our exclusion criteria warranted the absence of neurodevelopmental disorders, thus excluding children with ADHD from our experimental group; the attention subdomain yielded the poorest scores in the Ahmadi et al. study. It is important to note that our study found that both the total score and individual APDQ subdomains effectively differentiate clinical populations [16,38].

#### 4.2.2. APDQ Psychometric Properties

The APDQ psychometric properties reported here are in agreement with existing reports [16,38]. The Greek APDQ showed very good internal consistency comparable to that of the original English version and the Persian APDQ adaptation. Similarly, the current study’s intraclass classification coefficient Cronbach’s alpha (α = 0.926) was excellent. Our findings further document that the APDQ is a reliable and valid screening questionnaire for auditory processing difficulties in Greek Cypriot children.

#### 4.2.3. Correlation between CHAPS and APDQ with Auditory Processing Indices

Our findings indicate that children with SSDs perform lower than TD children on dichotic hearing tests, word discrimination in noise, and tests related to temporal processing, in agreement with the literature [10,39,40,41,42,43,44]. Shaikh, Baker, and Levya [40] reported significant correlations between CHAPS, DDT, and GIN. This underscores the potential value of screening tests to eliminate the necessity for further evaluation as well as the need to investigate the value of questionnaires as screening instruments for auditory processing difficulties in clinical populations. Furthermore, our study population aligns with the systematic review of Hearnshaw, Baker, and Munro [41], which indicated that most articles reported difficulties in speech perception tasks among preschool- and early-school-age children with SSDs. The persistence of these difficulties beyond the age of seven suggests a probable link to auditory processing issues, as highlighted in the systematic review by Drosos et al. [10] in children with SSDs. Therefore, it is crucial to identify distinguishing characteristics among populations and promptly refer individuals to an audiologist for accurate diagnosis, guidance for caregivers, and appropriate consultation with a rehabilitation specialist.

The current study contributes a valuable clinical perspective for the use of questionnaires to identify children with listening difficulties: we established that both the CHAPS and the APDQ have mostly moderate or strong correlations with Auditory Processing indices like duration and pitch patterns and reflect the listening difficulties in noise [22,41,42,43,44].

### 4.3. Limitations and Future Extensions

The relatively small sample size may present a limitation that lowers the applicability of our findings. Future research may involve a larger sample and inclusion of children with various comorbidities. A detailed follow-up of children referred for a comprehensive AP evaluation would strengthen the questionnaires’ value as screening instruments to identify children with listening difficulties.

## 5. Conclusions

This study is a preliminary validation of the Greek version of the CHAPS and APDQ in Greek Cypriot-speaking children. The questionnaires showed good validity and reliability, and our findings verified the psychometric properties of the tests and their clinical applicability for children with SSDs.

Validity findings for the CHAPS and the APDQ were consistent with those reported for other languages. As current practice indicates that children with APD are initially assessed by special educators, educational psychologists, and/or speech pathologists before a full diagnostic AP assessment, reliable instruments to identify children at risk for APD can be of tremendous help to the interdisciplinary team. The CHAPS and APDQ can identify children at risk for APD and facilitate early detection. They may guide the non-audiology specialists of the interdisciplinary team to appropriate referrals for children suspected of APD and other comorbidities and provide a valuable counseling basis to their families and caregivers.

It should be emphasized that questionnaires should not be used in isolation for APD diagnosis nor replace diagnostic protocols that include linguistic and non-linguistic tests and specialized audiological tests. These questionnaires are useful in assessing listening difficulties in an academic environment, especially in children with learning difficulties and persisting SSDs. Language and phonological awareness difficulties often resolve after treatment, whereas the AP challenges may persist and affect overall academic performance [8,18,32].

## Figures and Tables

**Table 1:** Sample characteristics and language performance.

	TD Group (N = 16)	Experimental Group (N = 24)		
	Mdn (IQR)	Mdn (IQR)	*p*	n^2^
Children’s Age in Years	8.40 (7.85–8.70)	8.10 (7.90–8.40)	0.486	0.007
Raven Total Score	33.00 (31.50–33.50)	32.00 (31.00–33.00)	0.153	0.004
Action Picture Test Total Score	71.00 (62.50–76.00)	68.00 (60.00–71.00)	0.170	0.102
Action Picture Test: information	37.00 (32.00–38.50)	33.00 (31.00–36.00)	0.930	0.007
Action Picture Test: grammar	34.00 (30.50–36.50)	30.00 (28.00–32.00)	<0.005 *	0.150
Metaphone Total Score	180.00 (166.50–187.50)	169.00 (163.00–175.00)	0.220	0.099
Words from Same Syllable Score	13.00 (12.00–13.00)	13.00 (12.00–13.00)	0.936	0.002
Syllabication Score	8.00 (8.00–8.00)	8.00 (8.00–8.00)	0.830	0.006
First Syllable Identification Score	12.00 (12.00–12.00)	12.00 (12.00–12.00)	0.936	0.000
Final Syllable Localization Score	24.00 (23.00–26.00)	24.00 (22.00–26.00)	0.630	0.006
Word Finding Syllable Criterion Score	8.00 (7.00–8.00)	8.00 (8.00–8.00)	0.708	0.006
First Phoneme Identification Score	27.00 (24.50–28.50)	23.00 (22.00–26.00)	<0.005 *	0.216
Word Finding Phoneme Criterion Score	15.00 (13.50–17.00)	14.00 (12.00–15.00)	0.099	0.071
Word Repetition Score	37.00 (34.50–39.50)	34.00 (32.00–37.00)	<0.005 *	0.075

Abbreviations: TD group, typical developing children; Mdn, Medians; IQR, interquartile range; * *p* level at *p* < 0.005; n^2^, partial eta squared.

**Table 2:** Auditory processing performance.

	TD Group (N = 16)	Experimental Group (N = 24)		
	Mdn (IQR)	Mdn (IQR)	*p*	n^2^
Gap Detection 500 Hz	10.00 (10.00–10.00)	10.00 (10.00–10.00)	0.147	0.053
Gap Detection 1000 Hz	10.00 (10.00–10.00)	10.00 (10.00–10.00)	=0.005	0.095
Gap Detection 2000 Hz	10.00 (10.00–10.00)	10.00 (10.00–10.00)	0.809	0.001
Gap Detection 4000 Hz	10.00 (10.00–10.00)	10.00 (9.25–10.00)	0.127	0.059
Gap Detection in Noise 1	7.00 (7.00–7.00)	7.00 (7.00–7.00)	0.147	0.053
Gap Detection in Noise 5	6.00 (5.00–7.00)	5.00 (2.25–5.75)	<0.005	0.195
Speech in Bubble Right Ear	64.50 (58.25–66.00)	54.00 (39.75–62.00)	<0.005	0.143
SNR 7	70.00 (60.00–78.75)	60.00 (51.25–73.75)	0.165	0.049
SNR 5	67.50 (55.00–70.00)	60.00 (45.00–73.75)	0.284	0.029
SNR 3	65.00 (55.00–70.00)	55.00 (40.00–63.75)	=0.005	0.093
SNR 1	62.50 (55.00–70.00)	60.00 (50.00–65.00)	0.151	0.052
SNR Minus 1	50.00 (36.25–55.00)	40.00 (20.00–45.00)	<0.005	0.156
SNR 50%	2.40 (1.85–2.95)	3.00 (2.25–4.40)	=0.005	0.092
Dichotic Words—Right Ear 1st	21.00 (21.00–22.00)	19.50 (18.25–21.00)	<0.001	0.336
Dichotic Words Synchronized	21.00 (20.00–22.00)	18.00 (16.25–21.00)	=0.001	0.264
Duration Pattern Sequence	30.00 (29.50–30.00)	28.00 (26.25–29.00)	<0.001	0.323
Pitch Pattern 1000 to 2000 Hz	8.00 (7.25–8.00)	7.50 (7.00–8.00)	0.067	0.086
Pitch Pattern 4000 to 6000 Hz	8.00 (8.00–9.00)	7.00 (7.00–8.00)	=0.001	0.269
Forward Digit Span	8.00 (7.00–9.00)	7.00 (7.00–8.00)	0.200	0.042
Backward Digit Span	6.00 (5.00–7.00)	5.00 (4.25–6.00)	<0.005	0.108

Abbreviations: TD group, typical developing children; Mdn, Medians; IQR, interquartile range; SNR, signal-to-noise ratio; *p* level at *p* < 0.005; n^2^, partial eta squared.

**Table 3:** Group performance and comparisons on the CHAPS questionnaire.

	Parents of Experimental Group Children (N = 24)	Parents of TD Children (N = 16)			
	Mdn (IQR)	Mdn (IQR)	Mann-Whitney U	*p*	n^2^
CHAPS Total score	−0.50 (−0.70–−0.15)	0.20 (−0.20–0.80)	38.500	<0.001	0.455
CHAPS Noise subdomain	−1.30 (−1.65–−0.70)	0.30 (−0.30–0.70)	16.000	<0.001	0.682
CHAPS Quiet subdomain	−0.40 (−0.65–0.00)	0.40 (0.00–1.00)	49.000	<0.001	0.387
CHAPS Ideal subdomain	−0.30 (−0.70–0.70)	1.00 (0.00–1.00)	61.500	<0.005	0.314
CHAPS Multiple Inputs subdomain	−0.30 (−0.70–0.700)	0.70 (0.00–1.00)	33.500	<0.001	0.526
CHAPS Auditory Memory Sequencing subdomain	−0.50 (−0.85–−0.20)	0.00 (−0.50–1.00)	73.500	<0.005	0.235
CHAPS Auditory Attention Span subdomain	−0.10 (−0.90–0.50)	0.30 (−0.10–0.80)	68.000	<0.005	0.364

Abbreviations: CHAPS, Children’s Auditory Performance Scale; Mdn, Medians; IQR, interquartile range; *p* level at *p* < 0.005; n^2^, partial eta squared.

**Table 4:** Group performance and comparisons on the APDQ questionnaire.

	Parents of Experimental Group Children (N = 24)	Parents of TD Children (N = 16)			
	Mdn (IQR)	Mdn (IQR)	Mann-Whitney U	*p*	n^2^
APDQ Total Score	95.00 (81.50–107.00)	79.50 (68.75–85.00)	49.000	=0.001	0.253
APDQ Auditory Processing subdomain	60.00 (54.75–68.25)	50.50 (44.25–55.25)	35.000	<0.001	0.340
APDQ Attention Control subdomain	18.00 (14.75–22.00)	15.50 (12.50–17.00)	79.000	<0.005	0.109
APDQ Language subdomain	20.00 (18.00–24.00)	17.50 (16.25–19.75)	58.000	<0.005	0.202
APDQ Auditory Processing Target subdomain	−42.00 (50.00–39.00)	−36.00 (37.00–31.25)	29.000	<0.001	0.383

Abbreviations: APDQ, Auditory Processing Domains Questionnaire; Mdn, Medians; IQR, interquartile range; *p* level at *p* < 0.005; n^2^, partial eta squared.

**Table 5:** ROC data analysis of the *CHAPS* total score and its 6 domains.

Subdomains	Cut-Off	AUC (95% CI)	*p*
CHAPS Total Score	−0.30	0.849 (0.715–0.983)	=0.001
CHAPS Noise	−0.60	0.937 (0.858–1.000)	<0.001
CHAPS Quiet	−0.20	0.808 (0.657–0.959)	<0.005
CHAPS Ideal	−0.80	0.759 (0.591–0.926)	<0.005
CHAPS Multiple Inputs	0.10	0.869 (0.745–0.993)	<0.001
CHAPS Auditory Memory Sequencing	−0.10	0.712 (0.528–0.896)	<0.005
CHAPS Auditory Attention Span	0.30	0.733 (0.556–0.910)	<0.005

Abbreviations: CHAPS, Children’s Auditory Performance Scale; AUC, area under curve; *p* level at *p* < 0.005.

**Table 6:** ROC data analysis of the APDQ total score and its 4 domains.

	Cut-Off	AUC (95% CI)	*p*
APDQ Total Score	90.00	0.820 (0.679–0.961)	<0.001 *
APDQ Auditory Processing subdomain	53.00	0.871 (0.753–0.989)	<0.001 *
APDQ Attention Control subdomain	18.00	0.710 (0.532–0.887)	<0.005 *
APDQ Language subdomain	18.00	0.785 (0.632–0.938)	=0.005 *
Auditory Processing Target subdomain	38.00	0.871 (0.753–0.989)	<0.001 *

Abbreviations: APDQ, Auditory Processing Domains Questionnaire; AUC, area under curve; * *p* level at *p* < 0.005.

**Table 7:** Correlations between the CHAPS and APDQ test findings and auditory processing performance (only significant findings are included in the table).

Parameters	CHAPS	APDQ
CHAPS		ρ = 0.639 **
APDQ	ρ = 0.639 **	
DPS		ρ = −0.522 **
DICHOTIC WORDS RE first	ρ = −0.354 *	
SNR − 1		ρ = −0.323 *
SNR 7	ρ = −0.392 **	
Pitch pattern 1000 to 2000 Hz	ρ = 0.256 **	
Pitch pattern 4000 to 6000 Hz		ρ = −0.316 *

Abbreviations: CHAPS, Children’s Auditory Performance Scale; APDQ, Auditory Processing Domains Questionnaire; DPS, Duration Pattern Sequence; RE, Right Ear, SNR, signal-to-noise ratio; ρ, Spearman’s rank correlation coefficient; *: *p* < 0.005; **: *p* < 0.001.

**Table 8:** Reported ROC curves of CHAPS across clinical populations.

	Cut-Off Ahmmed and Ahmmed, 2016 [34]	*p*	Cut-Off Sanchez and Lam, 2007 [36]	*p*	Cut-Off in Current Paper	*p*
CHAPS total			0.61	=0.124	−0.30	=0.001
CHAPS Noise subdomain			0.60	=0.248	−0.60	<0.001
CHAPS Quiet subdomain			0.63	=0.182	−0.20	<0.005
CHAPS Ideal subdomain	0.684	=0.932	0.50	=0.481	−0.80	<0.005
CHAPS Multiple Inputs subdomain			0.66	=0.124	0.10	<0.001
CHAPS Auditory Memory Sequencing subdomain	0.632	=0.016	0.66	=0.138	−0.10	<0.005
CHAPS Auditory Attention Span subdomain	0.697	=0.043	NR	NR	0.30	<0.005

Abbreviations: CHAPS, Children’s Auditory Performance Scale; NR, no report; *p* level at *p* < 0.005.

## Data Availability

The data presented in this study are available on request from the corresponding author due to ethical and privacy reasons.
